# The Effect of Some 4,2 and 5,2 Bisthiazole Derivatives on Nitro-Oxidative Stress and Phagocytosis in Acute Experimental Inflammation

**DOI:** 10.3390/molecules19079240

**Published:** 2014-07-02

**Authors:** Cătălin Araniciu, Alina Elena Pârvu, Mariana Doina Palage, Smaranda Dafina Oniga, Daniela Benedec, Ilioara Oniga, Ovidiu Oniga

**Affiliations:** 1 Department of Therapeutic Chemistry, Faculty of Pharmacy, “Iuliu Haţieganu” University of Medicine and Pharmacy, 12 Ion Creanga Street, 400010 Cluj-Napoca, Romania; E-Mails: araniciu.catalin@umfcluj.ro (C.A.); mpalage@umfcluj.ro (M.D.P.); smaranda.oniga@umfcluj.ro (S.D.O.); ooniga@umfcluj.ro (O.O.); 2 Department of Physiopathology, Faculty of Medicine, “Iuliu Haţieganu” University of Medicine and Pharmacy, 2-4 Victor Babes Street, 400010 Cluj-Napoca, Romania; 3 Department of Pharmacognosy, Faculty of Pharmacy, “Iuliu Haţieganu” University of Medicine and Pharmacy, 12 I. Creanga Street, 400010 Cluj-Napoca, Romania; E-Mails: dbenedec@umfcluj.ro (D.B.); ioniga@umfcluj.ro (I.O.)

**Keywords:** anti-inflammatory, nitro-oxidative stress, iNOS, bisthiazoles, antioxidant

## Abstract

Nineteen bisthiazoles were tested in order to assess their anti-inflammatory and antioxidant properties. First, we evaluated the *in vitro* direct antioxidant capacity of the bisthiazoles using the DPPH radical scavenging method. Then, the anti-inflammatory effect was tested in acute rat experimental inflammation by measuring the acute phase bone marrow response, the phagocytic capacity and the serum nitro-oxidative stress status. Although none of the substances showed significant direct antioxidant potential in the DPPH assay, most of them improved serum oxidative status, when administered to rats with inflammation. Four of the bisthiazoles proved to have good anti-inflammatory properties, similar or superior to that of equal doses meloxicam.

## 1. Introduction

Nonsteroidal anti-inflammatory drugs (NSAIDs) are one of the most frequently used therapeutic classes of drugs. Despite their large scale utilisation, many problems regarding their safety profile have been signalled over the years. Adverse reactions are numerous and frequent, stretching from gastro-intestinal problems to cardiovascular and renal events [1,2,3,4].

The quest for the optimal NSAID goes on as research now suggests that the COX-2 inhibition should be selective rather specific, as proved by the coxibe experience (only the less selective ones are still available for therapy) [5]. Furthermore, new NSAIDs act by simultaneously altering other pro-inflammatory mechanism, such as inhibiting iNOS. Due to the multiple connexions and similarities between COX-2 and iNOS, dual inhibitors could be more effective and better tolerated anti-inflammatory agents [6,7].

Considering that most of the selective COX-2 inhibitors that have been approved for market have a 1,2-di-aryl-heterocyclic structure, we decided to investigate the effects of compounds with similar structures. As a central moiety we focused on the bisthizole due to the presence of the thiazole ring in other NSAID (meloxicam) and due to bioisosterism of the thiazole with the pyrazole (celecoxib) or isoxazole ring (parecoxib). Also, our previous experience with anti-inflammatory thiazole compounds [8,9,10] encouraged us to investigate the activity of bisthiazole compounds.

In the same time, we decided to focuse on 1,3-diaryl-heterocyclic systems as recent studies showed that molecules with 1,3-diaryl-heterocyclic structures are also active as COX-2 inhibitors and may possess various central heterocyclic systems: pyrazole [11,12], triazole [13], tiazolidinedione [14].

In order to increase the selectivity for COX-2, which has a larger lipophilic pocket than COX-1, we focused on larger molecules, namely bisthiazoles with 1 or 2 aryl-substituents. Our aim was to assess the influence of a fourth ring presence in the molecule upon the anti-inflammatory activity. By evaluating nineteen bisthiazoles, belonging to two series (4,2 and 5,2 bisthiazoles) [15] with similar substituents, we also wanted to establish the correlation between the 3D shape of the molecule and its biological effect. The selected structural type could also act by inhibiting iNOS as suggested by results obtained for other thiazole-containing molecules [16,17]. Besides simultaneous inhibition of COX-2 and iNOS another potentially beneficial action in the treatment of inflammation could be the additional direct antioxidant effect of the molecule [18].

The aim of the study was to perform a complex anti-inflammatory activity profile of the synthesized bisthiazoles. Evaluation was done first *in vitro* through the determination of the direct antioxidant capacity by a DPPH radical scavenging method. Then, using an acute rat experimental inflammation model we assessed the acute phase bone marrow response, the phagocytic capacity and the nitro-oxidative stress.

## 2. Results and Discussion

### 2.1. Effects on the Acute Phase Bone Marrow Response

Table 1 summarises the effects of the tested bisthiazole derivatives on acute phase bone marrow response.

**Table 1:** The effects of the bisthiazoles on the acute phase bone marrow response.

Group	Leukocyte (no./mm^3^)	Neutrophils (%)	Monocytes (%)	Lymphocytes (%)
**Control**	4768.57 ± 389.12	54.43 ± 3.69	1.86 ± 0.90	23.43 ± 5.16
**Inflammation**	8840.00 ± 1227.44	72.00 ± 3.51	1.57 ± 0.79	23.14 ± 3.67
**Meloxicam**	4794.29 ± 669.05	62.14 ± 4.91	1.86 ± 1.07	21.14 ± 2.85
**1**	8057.50 ± 503.08	60.57 ± 2.23	1.71 ± 0.49	22.71 ± 3.50
**2**	8810.00 ± 653.57	68.57 ± 4.86	1.43 ± 0.79	19.14 ± 1.95
**3**	10,260.00 ± 522.11	72.43 ± 3.21	1.86 ± 0.90	18.86 ± 3.02
**4**	10,382.00 ± 649.36	76.00 ± 2.65	1.71 ± 0.95	20.43 ± 4.69
**5**	10,432.86 ± 656.93	78.17 ± 5.31	1.57 ± 0.53	22.86 ± 3.24
**6**	4684.00 ± 643.96	56.14 ± 4.74	1.43 ± 0.79	25.71 ± 4.19
**7**	6652.00 ± 945.79	68.67 ± 3.93	1.71 ± 0.76	25.43 ± 2.23
**8**	6725.00 ± 658.91	62.40 ± 3.85	1.57 ± 0.53	25.14 ± 2.73
**9**	6850.00 ± 771.36	71.71 ± 4.39	1.29 ± 0.49	22.14 ± 3.39
**10**	6367.14 ± 766.23	66.43 ± 6.40	1.71 ± 0.76	20.71 ± 2.36
**11**	3796.67 ± 738.12	52.00 ± 3.27	1.57 ± 0.79	23.43 ± 3.05
**12**	10,425.00 ± 693.42	67.57 ± 2.30	1.57 ± 0.79	22.71 ± 2.98
**13**	9677.50 ± 521.94	73.33 ± 7.31	1.86 ± 0.69	18.57 ± 1.51
**14**	9881.67 ± 794.06	72.80 ± 3.56	1.29 ± 0.49	24.29 ± 4.03
**15**	9995.00 ± 552.66	60.29 ± 3.9	1.57 ± 0.53	23.14 ± 4.88
**16**	3275.00 ± 861.68	52.00 ± 4.2	1.43 ± 0.79	22.86 ± 1.86
**17**	3922.86 ± 412.54	51.29 ± 4.5	1.71 ± 0.49	21.29 ± 5.25
**18**	3440.00 ± 716.87	49.86 ± 2.97	1.43 ± 0.53	18.00 ± 2.00
**19**	3037.14 ± 454.41	48.71 ± 2.98	1.57 ± 0.53	19.14 ± 2.19

All experiments were performed in triplicates. Results are expressed as means ± standard deviation.

When considering the total leukocyte count it was apparent that most of the tested compounds caused a decrease of this parameter, when compared with values obtained for the inflammation group (**I**). Compounds **6**, **7**, **8**, **9**, **10**, **11**, **16**, **17**, **18** and **19** had a statistically significant lowering effect (*p* < 0.01). The other tested compounds showed no important effect. By comparing the results obtained for the bisthiazoles compounds with those obtained for the meloxicam treated group (**M**) a number of five compounds proved to be significantly more potent than the reference NSAID meloxicam: compounds **11**, **16** and **17** (*p* < 0.05), compound **18** (*p* < 0.01) and compound **19** (*p* < 0.001). Also, compound **6** had a similar activity with meloxicam (*p* > 0.05).

By analysing the evolution of the neutrophils percentage a statistically significant reduction was seen for the compunds **1**, **6**, **11**, **15**–**19** (*p* < 0.001), **8** (*p* < 0.01) and **12** (*p* < 0.05), when comparing with the Inflammation group. Compounds **2**, **7**, **9** and **10** also decreased this parameter but in a non-significant manner (*p* > 0.05).

Six of the tested compounds showed a significantly better neutrophils percentage lowering effect than meloxicam (compounds **11**, **16**, **17**, **18**, **19** with *p* < 0.001 and **6** with *p* < 0.05), while compounds **1**, **8** and **15** had a similar activity as meloxicam.

In order to achieve a reduction of the inflammatory processes through the reduction of the cellular component of the inflammatory response, a substance must be able to induce a correlated decrease of the total leukocyte count and neutrophils percentage. In this regard, the bisthiazoles derivatives **6**, **8**, **11**, **16**, **17**, **18** and **19** have been shown to diminish total leukocyte count especially through the reduction of the neutrophils, and compounds **1**, **12** and **15** decreased neutrophils percentage but did not cause a corresponding reduction of the total leukocytes count.

### 2.2. Effects on the in Vitro Phagocytosis Test

**Figure 1:**
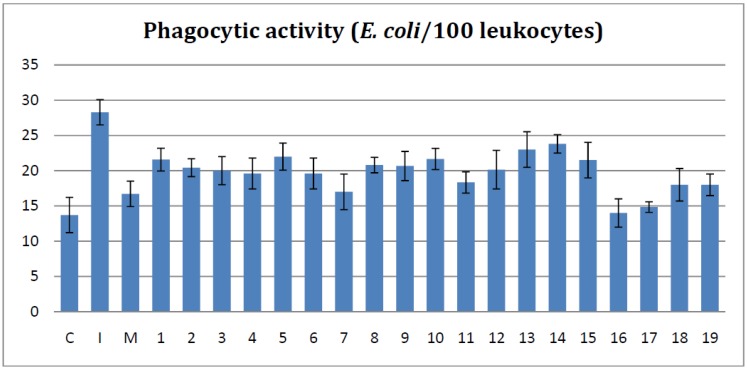
The effects of the tested bisthiazoles on the PA levels.

The first parameter use to quantify the phagocytosis was the phagocytic activity (PA) and its results are shown in Figure 1. When comparing the bisthiazoles groups with those of the Inflammation group, it was found that all compounds caused a rigorous and statistically significant decline of the PA values (*p* < 0.001). Moreover, when comparing with the meloxicam treated group, compounds **7**, **11**, **18**, and **19** showed a similar activities (*p* > 0.05). The compounds with the best reduction of PA values were the bisthiazoles **16** and **17** that proved to have a significantly better activity than meloxicam (*p* < 0.05).

C = negative control; I = inflammation; M = meloxicam.

**Figure 2:**
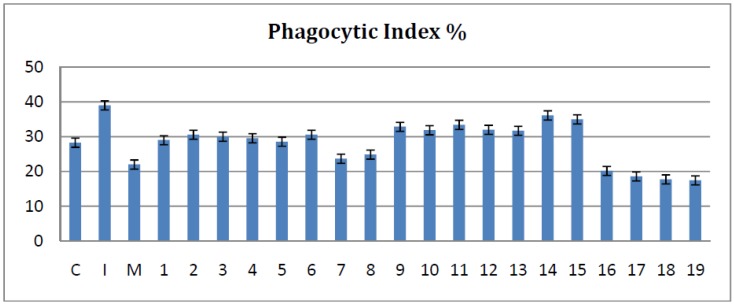
The effects of the tested bisthiazoles on the PI%.

When comparing to the inflammation group all tested compounds reduced the PI%. Most of the bisthiazoles showed a significant PI% reduction (*p* < 0.001), apart from compounds **14** and **15** that had a modest activity (*p* > 0.05) (Figure 2). When compared with the meloxicam treated group, compounds **16**, **17**, **18** and **19** showed a significantly more pronounced reduction of PI% (*p* < 0.05) and compounds **7** and **8** had a similar effect with meloxicam (*p* > 0.05).

C = negative control; I = inflammation; M = meloxicam.

### 2.3. Effects on the Serum Nitrites and Nitrates Levels

**Figure 3:**
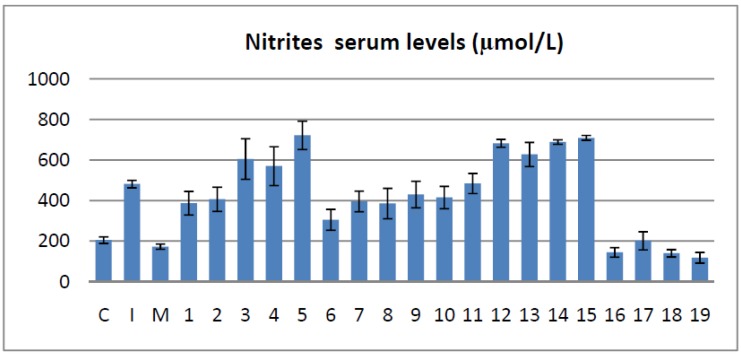
The effects of the tested bisthiazoles on the serum nitrites and nitrates levels.

The determination of serum nitrites and nitrates levels were used in order to measure the NO synthesis, which is known to be directly proportional to the intensity of the inflammatory process. NO hyper-production is mediated by iNOS and can be assessed indirectly by measuring NO metabolites nitrites and nitrates (NOx). Results of this assay are presented in Figure 3.

C = negative control; I = inflammation; M = meloxicam.

Comparing the tested compounds with the inflammation group the serum levels of NOx were significantly reduced by compounds **1**, **2**, **6**, **7**, **8** (*p* < 0.05) and compounds **16**, **17**, **18**, **19** (*p* < 0.001). The other bisthiazoles appeared to influence in a more modest manner NO production (compounds **9**, **10**) or even favoured the increase of NOx (compounds **3**–**5** and **11**–**15**).

When compared with the **M** group, four of the tested molecules had encouraging results: **17** had a similar activity with meloxicam, while **16** and **18** were significantly superior with *p* < 0.05. The best compound, for reducing NO production, seemed to be compound **19** that had a superior activity compared to meloxicam (*p* < 0.001).

### 2.4. Serum Oxidative Stress Evaluation

The evaluation of oxidative stress consisted of measuring the oxidant potential of the serum (as reflected by serum total oxidant status TOS) and the antioxidant potential of the serum (characterized by serum total antioxidant response TAR). In order to have a comprehensive view of the serum oxidative stress, oxidative stress index (OSI) was calculated.

#### 2.4.1. Effects on the Serum Total Oxidant Status

The serum TOS is a marker of the inflammatory process. The oxidative capacity is directly linked with the presence in the serum of reactive oxygen (ROS) and nitrogen species (RNS). These species are produced by the cells involved in inducing and/or maintaining inflammation.

**Figure 4:**
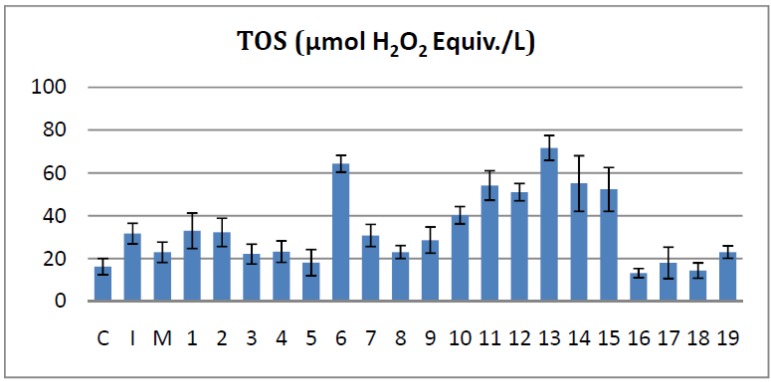
The effects of the tested bisthiazoles on the serum total oxidant status.

TOS levels were reduced, when compared with the Inflammation group, by compounds **8**, **16**, **18** (*p* < 0.001) and by compounds **3**, **4**, **5**, **17**, **19** (*p* < 0.01). The other tested molecules had TOS levels similar to the **I** group (**1**, **2**, **7**, **9**), or higher than that of the **I** group (**6**, **10**–**15**). Analyzing the potency of the active compounds by comparing them with meloxicam we ascertained that bisthiazoles **16** and **18** were significantly more potent in reducing TOS levels (*p* < 0.01). In the same time compounds **3**, **4**, **5**, **8**, **17** and **19** showed a similar TOS reducing activity with that of meloxicam (*p* > 0.05), as presented in Figure 4.

TOS = total oxidant status, C = negative control; I = inflammation; M = meloxicam.

#### 2.4.2. Effects on the Serum Total Antioxidant Response

Serum TAR is a parameter that characterises the antioxidant potential of the rat serum. Any increase in TAR, by comparison with the Inflammation group, can be correlated with a decrease of oxidative stress.

**Figure 5:**
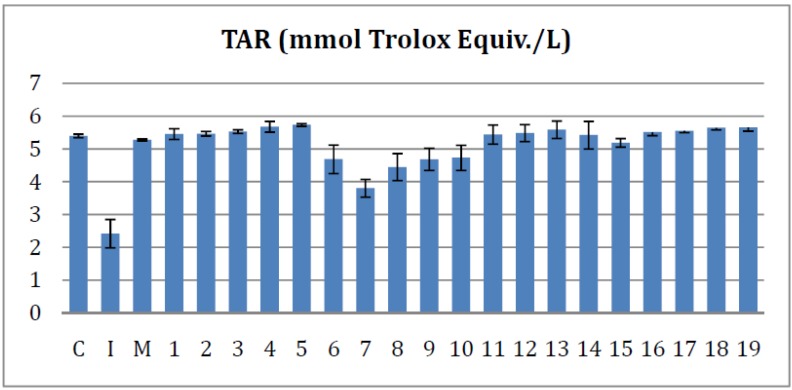
The effects of the tested bisthiazoles on the serum total antioxidant response.

As seen in Figure 5, all tested compounds appear to offered a significant increase of the antioxidant properties compared to the Inflammation group (*p* < 0.001). When compared to the effects of meloxicam, the bisthiazoles **2**–**5** and **16**–**19** showed a significantly improved ability to improve TAR values (*p* < 0.001) and compounds **1** and **13** had just a little better effect (*p* < 0.05). In the same time, compounds **11**, **12**, **14** and **15** had a similar effect to meloxicam on TAR. Compounds **6**, **7**, **8**, **9** and **10** effects on TAR were inferior to that of equal meloxicam doses (*p* < 0.05).

TAR = total antioxidant reactivity, C = negative control; I = inflammation; M = meloxicam.

#### 2.4.3. Effects on the Serum Oxidative Stress Index

**Figure 6:**
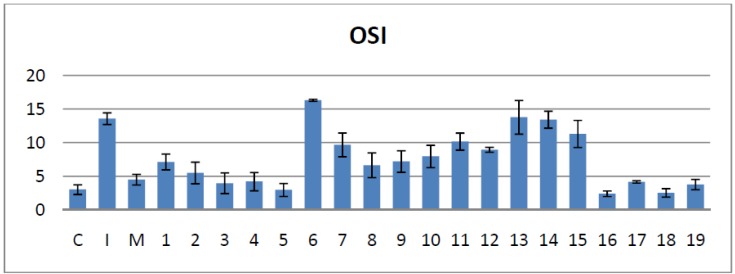
The effects of the tested bisthiazoles on the serum oxidative stress index.

OSI can provide a more pertinent view on serum oxidative stress levels. OSI calculations showed that most of the bisthiazoles derivatives tested can significantly improve serum oxidative status, when compared to the **I** group (*p* < 0.05), except compounds **13**, **14** and **15** which did not modify significantly OSI (*p* > 0.05) and compound **6** that actually increased oxidative stress (*p* < 0.01) (Figure 6).

Relative to meloxicam, compounds **3**, **5**, **16**, **18** and **19** have been proven to additionally reduce OSI (*p* < 0.05). The bisthiazoles **4** and **17** had a similar effect to meloxicam in improving the serum oxidative status (*p* > 0.05).

OSI = oxidative stress index, C = negative control; I = inflammation; M = meloxicam.

### 2.5. Direct Antioxidant Effect-DPPH Radical Scavenging Assay

The direct antioxidant effect of the new bisthiazoles was determined in order to assess if an eventual anti-inflammatory effect through the reduction of the oxidative stress may be due to the direct radical scavenging ability. A good free radical scavenging activity could be an asset in an AINS drug, as it suggests the potential of the compound to directly inactivate ROS and RNS, as part of the anti-inflammatory effects. The anti-scavenging ability was expressed using IC_50%_ values, and is presented in Table 2.

**Table 2:** The concentration of compound needed to scavenge 50% of the DPPH (IC_50%_) expressed in µg/mL.

Co.	1	2	3	4	5	6	7	8	9	10	11
IC_50%_	227.7	716.5	826.3	905.	192.01	1059.32	>1000	256.1	276.48	284.19	855.19
**Co.**	**12**	**13**	**14**	**15**	**16**	**17**	**18**	**19**	**Ascorbic Acid**	**BHT**	
IC_50%_	120.3	275.3	865.7	370.6	380.1	275.7	310.1	305.1	7.4	16.4	

All experiments were performed in triplicates and results are expressed as means, standard deviations were <5%.

The direct antiradical activity is strictly dependent on each structure. The differences in IC_50%_ values that were observed in compounds **1**–**7** and **8**–**19** showed that neither of the 2 structural scaffolds assures a good activity. By comparing the values obtained for analogues compounds (compounds with the same substituents but that belong to different series) it can be observed that the direct antioxidant effect is similar for the pairs 1/8, 3/11, and 5/13 but quite different for the others. Thus, it can be concluded that the presence of a certain scaffold or a certain substituent did not prove sufficient to induce similar antioxidant effects.

As it can be observed from the table above most of the tested compounds had no significant direct antioxidant activity. Thus, any improvement in the oxidant status of the serum of animals treated with the bisthiazoles was most likely due to other anti-inflammatory mechanisms, and not due to direct scavenging activity on the ROS and the RNS overproduced in the inflammatory status.

### 2.6. Overall Anti-Inflammatory Activity

The compounds from the 4,2-bisthiazoles series (compounds **1**–**7**) have generally showed a modest overall anti-inflammatory activity. Most of them do not cause a significant reduction of neutrophils percentage and/or total leukocyte count. The notable exception was compound **6** that caused a decline in neutrophil percentage more significantly than the reference meloxicam. Regarding the total serum oxidative stress, excepting **6**, most of the compounds caused its reduction. For the compounds **3**, **4** and **5** this has proved to be at least as good as that obtain for meloxicam. All compounds in this series significantly reduced PA and PI, while NO production was decreased just by compounds **1**, **2**, **6** and **7**. Considering these aspects we can conclude that for compounds **1**, **2** and **7** the decrease in phagocytosis could be correlated with a decrease in serum oxidative stress and NO production, while for compounds **3**, **4** and **5** it only correlated with a reduction of serum oxidant stress. The ability of compound **6** to decrease phagocytosis could be correlated just with a reduction of NO production.

The 5,2-bisthiazoles showed a varied anti-inflammatory activity. NO production was decreased by compounds **8** and **16**–**19**. Most compounds reduced OSI and improved serum oxidative status, except **13**, **14** and **15**. All 5,2-bisthiazoles reduced PA and most of them also reduced PI (except **14** and **15**).

Compounds **16**–**19** proved to have an activity significantly superior or at least equal to that of meloxicam and also correlated all tested anti-inflammatory mechanisms.

A good activity was also noticed for compound **8**. This compound showed good correlations between the various activities but did not proved to be comparable to meloxicam. Future studies should be performed in order to assess the toxicological profile of the compound. If toxicity studies will reveal a good tolerance for this compound, further studies, using higher doses should be performed in order to better characterize the anti-inflammatory activity.

Analysing the activity of analogues compounds from the two series, 4,2-bisthiazoles and 5,2-bisthiazoles, showed that the anti-inflammatory potential is dependent on the conformation of each molecule and no conclusion can be drawn as to which of the two scaffolds are more beneficial. When comparing analogue pairs results are mixed, with better activity being observed sometimes for the 4,2-bisthiazole analogues and other times for the 5,2-bisthiazole analogue.

From the point of view of the initial structural hypothesis, the results showed that a very good anti-inflammatory activity can be observed for the tricyclic compounds and that adding an extra nucleus proved to be detrimental for the biological activity. This was inferred by the fact that the four most active compounds, **16**–**19**, are the tricyclic ones. The importance of the tricyclic scaffold can be easily observed by comparing compound **15** and compound **16**. Replacing the the *p*-chlorophenyl in compound 15 with a chloromethyl group in compound **16** lead to a significant increase in the anti-inflammatory activity of the tricyclic compound. This finding is consistent to our previous results [10] that established that tricyclic thiazole compounds have a superior anti-inflammatory effect when compared to bicyclic ones.

Compounds **18** and **19** are 5,2-bisthiazoles that also include an ester group that could generate a free carboxylic group necessary for NSAID. From this point of view, these compounds could be acting as pro-drugs. In the same time, compound **17** contains a methyl-ketone that could be oxidized to the same carboxylic group, in a similar manner to nabumetone.

When considering the tetracyclic compounds, only **8** had a good anti-inflammatory activity, possible due to a smaller molecular volume assured by the lack of substituent on the nuclei.

These findings are consistent with the known SAR profile of NSAID [1,5] and also with data present by other researchers that investigated the diaryl-heterocyclic scaffold [11,12,13,14]. The replacement of the central heterocycle with a heterocyclic system (4,2- or 5,2-bisthiazole) lead to an increase in molecular volume, but also to an increase in molecular rigidity. As suggested by Kaur *et al.* [13], we also consider that a structural element that provides more molecular flexibility is required in order to obtain optimum effects.

## 3. Experimental

### 3.1. Chemistry

**Scheme 1:**
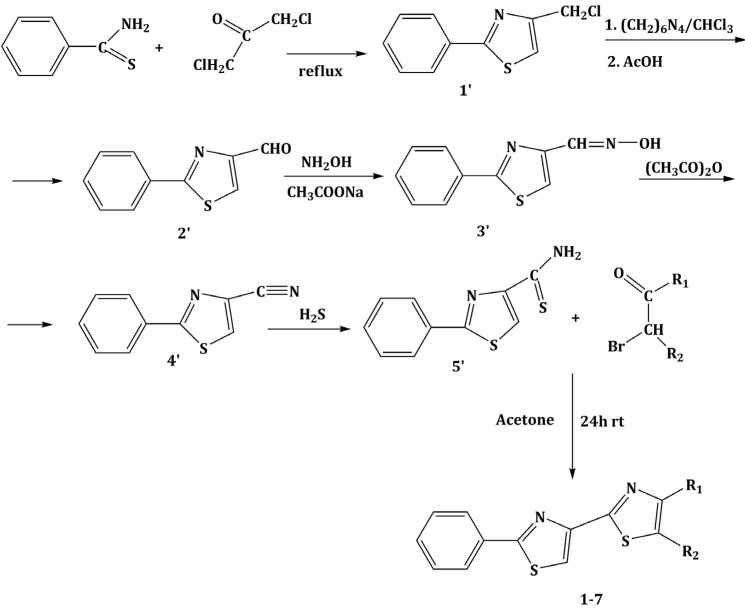
Reaction scheme for obtaining the 4,2-bisthiazoles **1**–**8** [15].

**Scheme 2:**
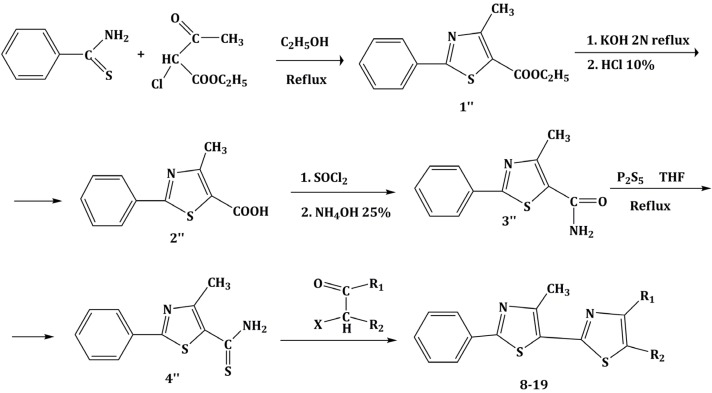
Reaction scheme for obtaining the 5,2-bisthiazoles **9**–**17** [15].

The chemical compounds were synthesized according to Scheme 1 and Scheme 2 [15] and characterized by our research team, as reported in our previous papers [15,19]. All molecules are bisthiazole compounds and belong to two structural types namely: 4,2-bisthiazole (compounds **1**–**7**) and 5,2-bisthiazole (compounds **8**–**19**). The chemical structures of the tested compounds are shown in Table 3.

**Table 3:** The bisthiazole compounds.

4,2-Bisthiazoles	5,2-Bisthiazoles	R_1_	R_2_
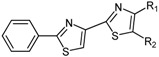	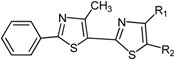		
**1**	**8**	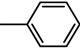	-CH_3_
**-**	**9**	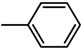	H
**2**	**10**	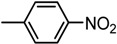	H
**3**	**11**	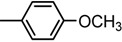	H
**4**	**12**	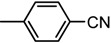	H
**5**	**13**	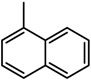	H
**6**	**14**	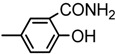	H
**7**	**15**	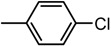	H
-	**16**	-CH_2_-Cl	H
-	**17**	-CH_3_	-CO-CH_3_
-	**18**	-CH_3_	-COOC_2_H_5_
-	**19**	-CH_2_-COOC_2_H_5_	H

The compounds were obtained through elaborate synthesis routes that comprised five, and six reactions, respectively. Both synthesis routes led to different key intermediates, of a thioamide nature, that were subsequently treated with the same α-haloketones in order to obtain derivatives with common structural moieties. The aim was to obtain similar 4,2- and 5,2-bisthiazole derivatives in order to better characterize the structure-activity relationship between the two structural types [15].

All chemical substances used to prepare the reagents needed in the quantitative determinations were of analytical grade purity and where purchased from Merck (Darmstadt, Germany) and Sigma-Aldrich (Taufkirchen, Germany).

### 3.2. Biological Evaluation

The evaluation of the anti-inflammatory activity was performed on rats by following an acute induced inflammation model. The anti-inflammatory activity was assessed by measuring the acute phase bone marrow response, phagocytes activity, oxidative stress status and NO synthesis. All methods were reported in our previous research [8,9,10,17].

#### 3.2.1. Animals

All procedures that involved the use of life animals followed the European guidelines and rules as established by the EU Directive 2010/63/EU. The study was conducted with the approval of the “Iuliu Hatieganu” University of Medicine and Pharmacy Institutional Animal Ethical Committee (no.31/10.02.2014).

Experiments were carried out on fully matured male albino rats (Wistar-Bratislava), weighing 200–250 g. The animals were obtained from the University of Medicine and Pharmacy Cluj-Napoca breeding facilities.

The study protocol involved the repartition of the animals in 21 groups consisting of ten individuals. The control group (**C**) consisted of healthy individuals that did not receive any substances. The inflammation group (**I**) received a pro-inflammatory substance administered i.m. (turpentine oil 0.6 mL/100 g body weight). Group **M** received the same inflammation inducing agent but also received a reference anti-inflammatory drug, meloxicam (doses at 3.2 mg/kg body weight). Groups **1**–**19** were treated with the pro-inflammatory turpentine oil and with the tested compounds **1**–**19** (equi-molar dose with meloxicam). The tested compounds were administered i.p. as suspensions in 1% carboxymethyl celullose in saline vehicle.

After treatment with the corresponding substances, all animals were maintained in a controlled environment for 24 h with food and water *ad libitum*, a 25 °C environment temperature and a 12 h light/dark cycle.

After 24 h, in order to reduce animal suffering, the subjects were anesthetized by i.p. administration of ketamine 90 mg/kg. Blood samples were collected by retro-orbital sinus puncture. Samples used for the acute phase bone marrow response and the *in vitro* phagocytosis test were harvested on EDTA, while samples used for serum separation were collected without anticoagulant. Serum separation was achieved by centrifugation at 1500 ×*g* for 10 min. All samples were tested immediately after harvesting.

#### 3.2.2. The Acute Phase Bone Marrow Response

The acute phase bone marrow response was assessed by determining the total leukocyte count and leukocyte count expressed as percentage [9,20].

#### 3.2.3. *In Vitro* Phagocytosis Test

The phagocytosis test was performed by incubating (at 37 °C, for 30 min) a blood sample harvested on EDTA with an *Escherichia coli* suspension (4 × 10^6^ germs/mL, in saline solution 0.9%). The mixture ratio was 0.2 mL blood/20 µL *E. coli* suspension. The count was made by Olympus optic microscope on smears stained May-Grunwald-Giemsa prepared from the incubation mixture.

Two distinct parameters were calculated to characterize the activity of phagocytes: the phagocytic activity (PA) defined as the number of *E. coli* germs phacocyted by 100 leukocytes and the phagocytic index (PI%) defined as the percentage of leukocytes that phagocyted at least one germ [9,21,22].

#### 3.2.4. Serum Nitric Oxide Synthesis Evaluation

Due to the fact that NO is a very reactive molecule, nitric oxide production was quantified indirectly by measuring the levels of total serum nitrite (NO_2_^−^) and nitrate (NO_3_^−^) (NOx). The procedure used was previously described in our work [10,23] and involves the Griess reaction. Serum samples were passed through 10-KD filters (Sartorius AG, Goettingen, Germany) and deproteinized by methanol/diethyl ether (3/1, v/v) (sample: methanol/diethyl ether, 1:9, v/v) [24]. To a 100 μL of serum sample an equal volume of VCl_3_ (8 mg/mL) was added, in order to reduce all nitrates to nitrites. Afterwards, 100 μL of Griess reagent (50 μL of SULF (2%) and 50 μL of NEDD (0.1%)) solution was added. The samples were then incubated for 30 min 37 °C, before reading the absorbance at 540 nm. Serum NOx was expressed as nitrite μmol/L [25].

#### 3.2.5. Oxidative Stress Evaluation

#### 3.2.5.1. Serum Total Oxidant Status Determination

Total oxidant status (TOS) of serum was determined using a colorimetric measurement method [17,26]. The method is based on the redox reaction between oxidant species, found in the serum, and the ferrous ion-*o*-dianisidine complex, which is oxidized to ferric ion. Glycerol molecules, present in the reaction medium, act as enhancers for the oxidation process. The resulting ferric ion is quantified due to its ability to form a colored complex with xylenol orange in an acidic medium that can be measured spectrophotometrically. The color intensity is directly proportional with the quantity of oxidants in the serum sample. The assay is calibrated with hydrogen peroxide (H_2_O_2_) and the results are expressed in μmol H_2_O_2_ Equiv./L.

#### 3.2.5.2. Serum Total Antioxidant Response Determination

The total antioxidant response is a measure of the serum antioxidant capacity and was determined via a colorimetric method. The method relies on the oxidation of the colorless o-dianisidine substrate to the bright yellowish-brown dianisyl radical, under the influence of the hydroxyl radical generated by the Fenton reaction. The initial addition of the serum sample leads to the partial inactivation of the hydroxyl radicals present in the reaction medium, due to the antioxidant properties of the serum. The total antioxidant capacity of the serum is reflected in its ability to prevent the color change. The assay is calibrated with Trolox and results are expressed as mmol Trolox Equiv./L [17,27].

#### 3.2.5.3. Calculation of Oxidative Stress Index

The oxidative stress index (OSI) is an indicator of the degree of oxidative stress obtained by taking into consideration both TAR and TOS simultaneously [17,28]. This involved establishing a percent ratio of the total oxidative status to the total antioxidant response. For this purpose, the result unit of TAR, mmol Trolox equivalent/L, was transformed to μmol Trolox equivalent/L and OSI was calculated with the formula: OSI (Arbitrary Unit) = TOS (μmol H_2_O_2_ Equiv./L)/TAC (mmol Trolox Equiv./L).

#### 3.2.6. Statistical Analysis

All results were expressed as mean ± standard deviation (SD). Statistical comparisons between the groups were made using Student’s *t* test. *p*-values < 0.05 were regarded as statistically significant. Pearson’s and Spearman’s correlation tests were performed in order to evaluate statistical correlation. Data was analyzed using the software: SPSS for Windows, version 16.

### 3.3. DPPH Radical Scavenging Assay

The direct antioxidant activity of the tested compounds was evaluated through a free radical scavenging assay namely the stable DPPH radical method. Samples were prepared by treating a series of methanolic 0.1 g/L DPPH solutions with an equal volume of the tested bisthiazole solution of different concentrations. Simultaneously, a control sample was prepared by diluting 1:1 the initial DPPH solution with methanol. The mixtures where incubated for 30 min at 40 °C and then the absorbance was measured at 517 nm.

A decrease in absorbance is associated with the reduction of the DPPH radical and thus directly proportional to the radical scavenging activity of the tested compounds. The DPPH scavenging ability was expressed as a percentage of absorbance reduction:

DPPH scavenging ability % = (Acontrol − A sample/Acontrol) × 100
(1)

Using DPPH scavenging ability % determined at different level of tested compounds concentrations, a curve was plotted in order to calculate the concentrations of compounds that leads to a 50% reduction of absorbance IC_50_. This is the concentration of compound needed to scavenge 50% of the free DPPH radical. As a positive control we used well known antioxidants including butylated hydroxytoluene (BHT) and ascorbic acid. For results interpretations we considered IC_50_ ≤ 50 µg/mL values as a high antioxidant capacity; 50 µg/mL < IC_50_ ≤ 200 µg/mL value were considered to reflect a moderate antioxidant capacity and IC_50_ > 200 µg/mL values were seen as no relevant antioxidant capacity [29,30,31].

## 4. Conclusions

Two series of bisthiazoles were tested in order to determine their anti-inflammatory potential and their *in vitro* radical scavenging activity. From a total of 19 compounds the primary anti-inflammatory mechanism, the reduction of total leukocyte count by lowering of the neutrophils percentage, was observed in compounds **6**, **8**, **11** and **16**–**19**. Compounds **8** and **16**–**19** correlated that with the reduction of the nitro-oxidative stress, with a potency and in a manner comparable or superior with that of the meloxicam. However, compound **6** did not reduce TOS, and compound **11** failed to reduce NO production and TOS. These results suggest that the tricyclic compounds are more active than tetracyclic derivatives.

The non-significant *in vitro* radical scavenging activity suggest that any improvement in the oxidant stress index of the serum is more likely caused through the anti-inflammatory mechanism that involves reducing phagocytic capacity and thus the levels of ROS and RNS exhibited by the tested compounds.

## References

[1] Ansari R., Castejon A.M., Cubbedu L., Fuller C., Gauthier T., Gazze D., Graham K.K., Moorman Li R., McLaughlin-Middlekauffe J., Motycha C., Harvey R. (2012). Anti-inflammatory Drugs and Autacoids. Lippincott’s Ilustrated Reviews: Pharmacology.

[2] Bhala N., Emberson J., Merhi A., Abramson S., Arber N., Baron J.A., Bombardier C., Cannon C., Farkouh M.E., FitzGerald G.A. (2013). Vascular and upper gastrointestinal effects of non-steroidal anti-inflammatory drugs: Meta-analyses of individual participant data from randomised trials. Lancet.

[3] Bäck M., Yin L., Ingelsson E. (2012). Cyclooxygenase-2 inhibitors and cardiovascular risk in a nation-wide cohort study after the withdrawal of rofecoxib. Eur. Heart J..

[4] García Rodríguez L.A., González-Pérez A., Bueno H., Hwa J. (2011). NSAID use selectively increases the risk of non-fatal myocardial infarction: A systematic review of randomised trials and observational studies. PLoS One.

[5] Friel C.J., Lu M.C., Beale B. (2011). Analgesisc. Wilson and Gisvold’s Textbook of Organic Medicinal and Pharmaceutical Chemistry.

[6] Kim S.F. (2011). The role of nitric oxide in prostaglandin biology; update. Nitric Oxide.

[7] Lim K.M., Lee J.Y., Lee S.M., Bae O.N., Noh J.Y., Kim E.J., Chung S.M,, Chung J.H. (2009). Potent anti-inflammatory effects of two quinolinedione compounds, OQ1 and OQ21, mediated by dual inhibition of inducible NO synthase and cyclooxygenase-2. Br. J. Pharmacol..

[8] Oniga S., Parvu A.E., Tiperciuc B.G., Palage M., Oniga O. (2011). The study of the anti-inflammatory activity of some thiazolyl-Δ_2_1,3,4 oxadiazolines and 5-carboxiethyl-2-hydrazon-4-methyl-thiazole derivatives. Farmacia.

[9] Moldovan M., Oniga O., Pârvu A.E., Tiperciuc B.G., Verite P., Pîrnău A., Crişan O., Bojiţă M., Pop R. (2011). Synthesis and anti-inflammatory evaluation of some new acyl-hydrazone bearing 2-aryl-thiazole. Eur. J. Med. Chem..

[10] Araniciu C., Parvu A.E., Tiperciuc B.G., Palage M., Oniga S., Verite P., Oniga O. (2013). Synthesis and evaluation of the anti-inflammatory activity of some 2-(trimethoxyphenyl)-4-R_1–5_-R_2_-thiazoles. Dig. J. Nanomater. Biostruct..

[11] Eren G., Unlü S., Nuñez M.T., Labeaga L., Ledo F., Entrena A., Banoğlu E., Costantino G., Sahin M.F. (2010). Synthesis, biological evaluation, and docking studies of novel heterocyclic diaryl compounds as selective COX-2 inhibitors. Bioorg. Med. Chem..

[12] El-Sayed M.A., Abdel-Aziz N.I., Abdel-Aziz A.A., El-Azab A.S., Asiri Y.A., Eltahir K.E. (2011). Design, synthesis, and biological evaluation of substituted hydrazone and pyrazole derivatives as selective COX-2 inhibitors: Molecular docking study. Bioorg. Med. Chem..

[13] Kaur J., Bhardwaj A., Sharma S.K., Wuest F. (2013). 1,4-Diaryl-substituted triazoles as cyclooxygenase-2 inhibitors: Synthesis, biological evaluation and molecular modeling studies. Bioorg. Med. Chem..

[14] Santin J.R., Uchôa F.D., Lima Mdo C., Rabello M.M., Machado I.D., Hernandes M.Z., Amato A.A., Milton F.A., Webb P., Neves Fde A. (2013). Chemical synthesis, docking studies and biological effects of a pan peroxisome proliferator-activated receptor agonist and cyclooxygenase inhibitor. Eur. J. Pharm. Sci..

[15] Araniciu C., Palage M., Oniga S., Pîrnău A., Verité P., Oniga O. (2013). Synthesis and characterization of some novel 5,2 and 4,2 bisthiazoles derivatives. Rev. Chim. Buchar..

[16] Ueda S., Terauchi H., Kawasaki M., Yano A., Ido M. (2004). Structure-activity relationships of 2-aminothiazole derivatives as inducible nitric oxide synthase inhibitor. Chem. Pharm. Bull. Tokyo.

[17] Tiperciuc B.G., Parvu A.E., Tamaian R., Nastasa C., Ionut I., Oniga O. (2013). New anti-inflammatory thiazolyl-carbonyl-thiosemicarbazides and thiazolyl-azoles with antioxidant properties as potential iNOS Inhibitors. Arch. Pharm. Res..

[18] Korbecki J., Baranowska-Bosiacka I., Gutowska I., Chlubek D. (2013). The effect of reactive oxygen species on the synthesis of prostanoids from arachidonic acid. J. Physiol. Pharmacol..

[19] Araniciu C., Marutescu L., Oniga S., Oniga O., Chifiriuc M.C., Palage M. (2014). Evaluation of the antimicrobial and anti-biofilm activity of some 4,2 and 5,2 bisthiazoles derivatives. Dig. J. Nanomater. Biostruct..

[20] Gougerot-Pocidalo M.A., Benna J., Elbim C., Chollet-Martin S., Dang M.C. (2002). Regulation of human neutrophil oxidative burst by pro- and anti-inflammatory cytokines. J. Soc. Biol..

[21] Hrabak A., Bajor T., Csuka I. (2006). The effect of various inflammatory agents on the alternative metabolic pathways of arginine in mouse and rat macrophages. Inflamm. Res..

[22] Hrabak A., Bajor T., Csuka I. (2008). The effect of various inflammatory agents on the phagocytosis and cytokine profile of mouse and rat macrophages. Inflamm. Res..

[23] Parvu A.E., Alb S.F., Craciun A., Taulescu M.A. (2013). Efficacy of subantimicrobial-dose doxycycline against nitrosative stress in chronic periodontitis. Acta Pharmacol. Sin..

[24] Ghasemi A., Hedayati M., Biabani H. (2007). Protein precipitation methods evaluated for determination of serum nitric oxide end products by the Griess assay. J. Med. Sci. Res..

[25] Miranda K.M., Espey M.G., Wink D.A. (2001). A rapid, simple spectrophotometric method for simultaneous detection of nitrate and nitrite. Nitric Oxide.

[26] Erel O. (2005). A new automated colorimetric method for measuring total oxidant status. Clin. Biochem..

[27] Erel O. (2004). A novel automated method to measure total antioxidant response against potent free radical reactions. Clin. Biochem..

[28] Harma M., Harma M., Erel O. (2003). Increased oxidative stress in patients with hydatidiform mole. Swiss Med. Wkly..

[29] Espin J.C., Soler-Rivas C., Wichers H.J. (2000). Characterization of the total free radical scavenger capacity of vegetable oils and oil fractions using 2,2-diphenyl-1-picrylhydrazyl radical. J. Agric. Food Chem..

[30] Benedec D., Vlase L., Oniga I., Mot A.C., Damian G., Hanganu D., Duma M., Silaghi-Dumitrescu R. (2013). Polyphenolic composition, antioxidant and antibacterial activities for two romanian subspecies of *Achillea distans* Waldst. et Kit. ex willd. Molecules.

[31] Chen Z., Bertin R., Froldi G. (2013). EC_50_ estimation of antioxidant activity in DPPH· assay using several statistical programs. Food Chem..

